# Penetration model for chemical reactivation for resin-embedded green fluorescent protein imaging

**DOI:** 10.1117/1.JBO.24.5.051406

**Published:** 2018-11-28

**Authors:** Longhui Li, Ruixi Chen, Xiuli Liu, Ning Li, Xiaoxiang Liu, Xiaojun Wang, Tingwei Quan, Xiaohua Lv, Shaoqun Zeng

**Affiliations:** a Huazhong University of Science and Technology, Britton Chance Center for Biomedical Photonics, Wuhan National Laboratory for Optoelectronics, Wuhan, Hubei, China; b Huazhong University of Science and Technology, Collaborative Innovation Center for Biomedical Engineering, School of Engineering Sciences, MoE Key Laboratory for Biomedical Photonics, Wuhan, Hubei, China

**Keywords:** penetration model, resin embedding, chemical reactivation, fluorescence imaging

## Abstract

In the so-called surface microscopy, serial block-face imaging is combined with mechanic sectioning to obtain volumetric imaging. While mapping a resin-embedded green fluorescent protein (GFP)-labeled specimen, it has been recently reported that an alkaline buffer is used to chemically reactivate the protonated GFP molecules, and thus improve the signal-to-noise ratio. In such a procedure, the image quality is highly affected by the penetration rate of a solution. We propose a reliable penetration model to describe the penetration process of the solution into the resin. The experimental results are consistent with the parameters predicted using this model. Thus, this model provides a valuable theoretical explanation and aids in optimizing the system parameters for mapping resin-embedded GFP biological samples.

To obtain volumetric imaging of biological tissues effectively, serial block-face imaging combined with mechanic sectioning is performed.^1^^–^^5^ After a block-face image is taken, an ultrathin slice of the specimen surface is cut off with a knife, and then, the next block-face image is taken. By repeating the above operation, a three-dimensional (3-D) atlas is obtained.

The recently reported chemical reactivation (CR) method, which can recover quenched fluorescence of green fluorescent protein (GFP) molecules, is very important to ensure the image quality of a resin-embedded GFP-labeled specimen.^6^ In the CR method, an alkaline buffer penetrates into the specimen, and then, the GFP fluorophores at the surface layer of the specimen are reactivated; simultaneously, the unprocessed nether layers remain in the quenched state.^7^ In such a procedure, the signal-to-noise ratio of the block-face fluorescence imaging of the specimen is improved,^8^^,^^9^ and the image quality is highly affected by the penetration rate of the alkaline buffer. Therefore, quantifying the penetration rate is urgently required.

In this paper, we propose a reliable penetration model to quantitatively describe the penetration process. Using a homogeneous and isotropic sample, we obtain the penetration data of the alkaline buffer; then, based on this data and the mass transfer theory, we constructed a penetration model to quantify the penetration rate in a resin-embedded GFP-labeled biological sample and obtain the curves of the CR thickness (that is reactivated fluorescence depth) that increases with time.

An ideal homogeneous and isotropic specimen (standard sample) was prepared to obtain the penetration data of the alkaline buffer. The fluorescein isothiocyanate (FITC) dye has been demonstrated to have CR properties similar to the GFP, and it dissolves uniformly in the resin;^10^ therefore, we choose FITC as a fluorescent tracer to demonstrate the penetration process. We dissolved $10^{-5}$ mol/L FITC dye in the glycol methacrylate (GMA Technovit 8100, Electron Microscopy Sciences) resin to polymerize into a standard sample. The fluorescence of the FITC fluorophores in the standard sample was significantly quenched during embedding process. When the alkaline buffer (${\mathrm{Na}}_{2}{\mathrm{CO}}_{3}$ solution, pH=10) drops on the surface, the ${\mathrm{OH}}^{-}$ ions transfers through the micropores formed in GMA resin polymer and then reactivates the FITC fluorophores immediately.^10^ The chemical reaction occurs in milliseconds,^11^ so the reactivation speed of quenched FITC was dominated by the transfer speed of ${\mathrm{OH}}^{-}$ ions, and the latter was determined by the diffusion coefficient of the sample.^12^^,^^13^ Since the recovered fluorescence intensity increases as the concentration of the penetrated ${\mathrm{OH}}^{-}$ increases,^6^^,^^10^ the penetration speed and distribution could be visualized by 3-D fluorescence imaging.

The penetration data of the standard sample are acquired by a confocal microscopy (LSM780, Zeiss) by performing continuous 3-D scanning imaging, and the results are shown in Fig. 1. The 3-D image contains 50 layers of two-dimensional images (x y planes), the step size in z direction for sampling is 1  μm, 50  μm in total (10  μm above the surface of the standard sample and 40  μm below it). The recovered fluorescence intensity in the xy planes at different times shows that the standard sample is homogeneous and isotropic [Fig. 1(a)]. According to the fluorescence intensity distribution in the xz and yz planes [Figs. 1(b) and 1(c)], the thickness of fluorescence intensity increases with increasing penetration time, and a gradient can be observed along the z axis, which demonstrates that the penetration of the alkaline buffer could be considered as a one-dimensional semi-infinite mass transfer process.^13^ We calculated the mean value of all the pixels in the xy planes to represent the fluorescence intensity. The normalized fluorescence intensity distribution curves along the z axis at different penetration times are shown in Fig. 1(d). The zero-coordinate position represents the surface of the standard sample, where the fluorescence intensity within a certain distance from the surface reaches its maximum (value of 1); this area is called the saturated region. After the saturated region, the fluorescence intensity drops sharply to the background (value of 0), and this area is called the unsaturated region. The fluorescence intensity at z=0 has negligible changes (<4%) in 650 s [Fig. 1(e)], because we chose very low laser power to avoid the fluorescence quenching, and the fluorophores embedded in the resin cannot diffuse freely. The curves in Fig. 1(f) show the penetration distance increasing with the square root of time. The measured data are on the isointensity contours of $I_{0.5}$ and $I_{0.9}$, respectively. The blue-dashed lines fit well with the measured data, and the determination coefficients (a parameter to judge the degree of fit; its value ranges from 0 to 1; and the larger the value, the better the fit degree) are 0.981 and 0.996, respectively. The penetration distance of the given fluorescence intensity (or the given concentration of the alkaline buffer) is proportional to the square root of time, which conforms to Fick’s second law.^13^^,^^14^

**Fig. 1:**
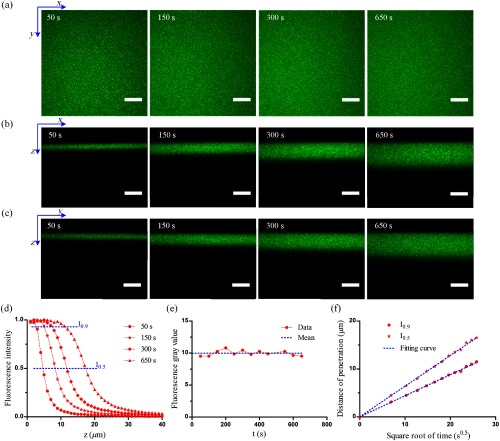
Penetration data of the standard sample. (a)–(c) Fluorescence images in the xy, xz, and yz planes of the standard sample at different times, respectively. (d) Normalized fluorescence intensity distribution curves of the standard sample along the z axis at different penetration times. As the penetration time increases, the penetration distance increases significantly. $I_{0.5}$ and $I_{0.9}$ (blue-dashed line) denote isointensity contours of 0.5 and 0.9, respectively. (e) Fluorescence gray value at z=0 at different times. The blue-dashed line is mean value. (f) The curves show the penetration distance increasing with the square root of time. The measured data are on the isointensity contours of $I_{0.5}$ and $I_{0.9}$, respectively. The blue-dashed lines are linear fitting curves and the determination coefficients are 0.997 and 0.998, respectively. Scale bars in (a) and (b): 10  μm.

The diffusion coefficient of the standard sample is a constant, and Fick’s second law is^14^

$$\frac{\partial C_{t,z}}{\partial t}=D\frac{\partial^{2}C_{t,z}}{\partial z^{2}},$$ (1)

where D is the diffusion coefficient (${\mu m}^{2}/s$), z denotes the penetration distance (μm), t denotes the penetration time (s), and $C_{t,z}$ is the concentration of ${\mathrm{OH}}^{-}$ ($\mathrm{mol}/{\mu m}^{3}$).

To maintain surface of the sample at a constant concentration (normalized value of 1), with the initial concentration being 0 throughout the sample, the analytical solution of Eq. (1) satisfies the initial condition [Eq. (2)] and the boundary condition [Eq. (3)]:

$$C_{t,z}=0,t=0,z>0,$$ (2)

$$C_{t,z}=1,t>0,z=0.$$ (3)

Through the derivation,^14^ the general mathematical expression of the penetration model is expressed as

$$C_{t,z}=\mathrm{erfc}(\frac{z}{2\sqrt{Dt}}),$$ (4)

where erfc is the complementary error function.

Once the alkaline buffer penetrates into the sample, the protonated and quenched FITC fluorophores are ionized immediately and converted into a fluorescent dianion state. Owing to the very low concentration of FITC in the standard sample, the amount of ${\mathrm{OH}}^{-}$ loss caused by the deprotonation reaction is negligible. Considering the existence of saturation, we use a factor K (>1) to obtain the relationship between the concentration of dianion $C(F^{2-})$ and the penetrated ${\mathrm{OH}}^{-}$ concentration $C_{t,z}$:

$$C(F^{2-})=\{\begin{array}{ll} C{\left(F^{2-}\right)}_{\max} & \text{where }C_{t,z}\in [1/K,1]\\ C{\left(F^{2-}\right)}_{\max}\cdot K\cdot C_{t,z} & \text{where }C_{t,z}\in [0,1/K] \end{array}.\;$$ (5)

In this equation, the factor K determines the extent of the saturated region, therefore, it is called the saturation coefficient. FITC ionizes almost completely in the saturated region, therefore, the concentration of the dianion $C(F^{2-})$ reaches a maximum $C{\left(F^{2-}\right)}_{\max}$. In contrast, in the unsaturated region, the concentration of the dianion is proportional to the penetrated ${\mathrm{OH}}^{-}$ concentration $C_{t,z}$.

The relationship between the recovered fluorescence intensity $I_{f}$ and concentration of the dianion $C(F^{2-})$ is given by^15^

$$I_{f}=\alpha_{f}\cdot I_{0}\cdot C(F^{2-}).$$ (6)

In this equation, $I_{0}$ denotes the excitation intensity and $\alpha_{f}$ is a constant consisting of the quantum efficiency of the dianion and instrument-related factors.

After substituting Eqs. (4) and (5) into Eq. (6), and normalizing the equation, the fluorescence intensity distribution of CR based on the penetration model is expressed as follows:

$$I_{f}=\{\begin{array}{ll} 1 & \text{where }C_{t,z}=\mathrm{erfc}(\frac{z}{2\sqrt{Dt}})\in [1/K,1]\\ K\cdot \mathrm{erfc}(\frac{z}{2\sqrt{Dt}}) & \text{where }C_{t,z}=\mathrm{erfc}(\frac{z}{2\sqrt{Dt}})\in [0,1/K] \end{array}.$$ (7)

Equation (7) is the general mathematical expression of the fluorescence intensity distribution of CR, in which the diffusion coefficient D and saturation coefficient K can be calculated according to the penetration data. Here, we select the data on the isointensity contours of $I_{0.5}$ and $I_{0.9}$ in the unsaturated region to calculate D and K of the standard sample. The slopes of the linear fitting curves in Fig. 1(d) are 0.642 and 0.441, respectively. Then, the following two equations can be obtained:

$$K\cdot \mathrm{erfc}(\frac{0.642}{2\sqrt{D}})=0.5,$$ (8)

$$K\cdot \mathrm{erfc}(\frac{0.441}{2\sqrt{D}})=0.9.$$ (9)

By solving these equations, we obtain the values D=0.138 and K=2.233. Substituting them into Eq. (7), the exact expression of the fluorescence intensity distribution of CR is obtained as

$$I_{f}=\{\begin{array}{ll} 1, & \text{where }C_{t,z}=\mathrm{erfc}(\frac{z}{0.744*\sqrt{t}})\in [0.45,1]\\ 2.233\cdot \mathrm{erfc}(\frac{z}{0.744*\sqrt{t}}), & \text{where }C_{t,z}=\mathrm{erfc}(\frac{z}{0.744*\sqrt{t}})\in [0,0.45] \end{array}.\;$$ (10)

Next, we verify the reliability of the penetration model. As shown in Fig. 2(a), the fluorescence intensity distribution curves of CR calculated by Eq. (10) are consistent with the measured ones. The determination coefficients are 0.975 (50 s), 0.985 (150 s), 0.993 (300 s), and 0.966 (650 s), respectively. It proves that the constructed penetration model is very reliable to accurately quantify the penetration rate. The corresponding normalized concentration distribution curves of the hydroxyl ion calculated by the penetration model at different penetration times conform to Fick’s second law [Fig. 2(b)]. According to the saturation coefficient K (2.233), the block-face of the sample where $C_{t,z}$ is greater than 0.45 (1/K=0.45, red-dashed line) is the saturated region of the fluorescence intensity; the nether layers of the sample where $C_{t,z}$ is less than 0.45 is the unsaturated region of the fluorescence intensity.

**Fig. 2:**
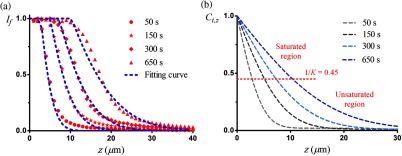
Verification of the penetration model reliability. (a) The fluorescence intensity distribution curves calculated by the penetration model (blue-dashed line) are consistent with the measured data (red symbols). (b) The normalized concentration distribution curves of a hydroxyl ion at different penetration times, which conforms to Fick’s second law. The block-face of the sample where $C_{t,z}$ is greater than 0.45 (1/K=0.45, red-dashed line) is the saturated region of the fluorescence intensity, and the nether layers of the sample where $C_{t,z}$ is less than 0.45 is the unsaturated region.

As mentioned above, based on Fick’s second law and the penetration data, a reliable penetration model was constructed successfully. To confirm its applicability, we further used the model to describe the penetration process in a resin-embedded GFP-labeled tissue.

Animal care and use were in accordance with the guidelines of the Administration Committee of Affairs Concerning Experimental Animals in Hubei Province of China. The protocol was approved by the Committee on the Ethics of Animal Experiments of the Huazhong University of Science and Technology. Following the protocol described by Gang et al.,^7^ a Thy1-EGFP mouse brain was embedded in Lowicryl HM20 (Electron Microscopy Sciences) resin. The CR results acquired by the confocal microscopy (LSM780, Zeiss) are shown in Fig. 3. The step size in z direction for sampling is 0.5  μm, 25  μm in total. The hippocampus with dense nerve fibers was chosen as the imaging region. Before CR, this region showed very weak fluorescence [Fig. 3(a)] because the resin-embedding process had quenched the GFP molecules. The fluorescence intensity of the nerve fibers was dramatically enhanced after CR and the fine structures of nerve fibers reappeared [Fig. 3(b)]. The CR thickness increased with time, and there was a gradient of fluorescence intensity along the z axis [Fig. 3(c)]. Since the fluorescence intensity is uniform only along the nerve fiber, we calculated the mean value of pixels for a single nerve fiber in the xy planes to represent its fluorescence intensity, 10 nerve fibers in total. The normalized fluorescence intensity distribution curves along the z axis at different times are shown in Fig. 3(d). Unlike the standard sample, the GFP sample had slight background fluorescence (value of 0.1) to be subtracted. The curves in Fig. 3(e) show that the CR thickness increases with the square root of time. The slopes of the fitted curves are 0.210 and 0.106, respectively. Through calculation, we obtain D=0.026, K=1.404, and the exact expression of the CR fluorescence intensity distribution is shown as follows:

$$I_{f}=\{\begin{array}{ll} 1, & \text{where }C_{t,z}=\mathrm{erfc}(\frac{z}{0.322*\sqrt{t}})\in [0.71,1]\\ 1.404\cdot \mathrm{erfc}(\frac{z}{0.322*\sqrt{t}}) & \text{where }C_{t,z}=\mathrm{erfc}(\frac{z}{0.322*\sqrt{t}})\in [0,0.71] \end{array}.\;\;$$ (11)

**Fig. 3:**
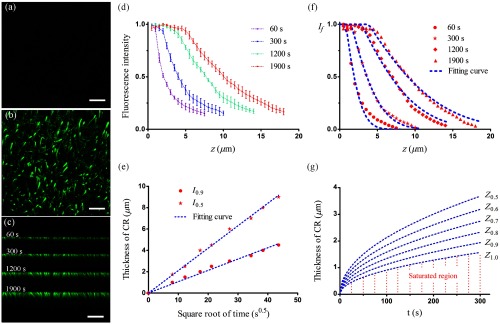
Penetration model in Lowicryl HM20 resin-embedded Thy1-EGFP mouse brain. (a) and (b) Maximum value projection of the 3-D images in the xy plane before and after CR, respectively. (c) Maximum value projection of the 3-D images in the xz plane at different times. (d) Normalized fluorescence intensity distribution curves along the z direction at different times. (e) The curves show the CR thickness increasing with the square root of time. The blue-dashed lines are linear fitting curves and the determination coefficients are 0.995 and 0.988, respectively. (f) The fluorescence intensity distribution curves calculated by the penetration model (blue-dashed line) are consistent with the measured ones after subtracting the background fluorescence intensity (red symbols). (g) The curves calculated by the penetration model indicate CR speed and fluorescence intensity gradient at different times. Scale bar: (a) and (b) 30  μm; (c) 20  μm.

Figure 3(f) shows that the fluorescence intensity distribution curves calculated by Eq. (11) (blue-dashed lines) are consistent with the normalized mean fluorescence intensity after subtracting the background fluorescence intensity (red symbols). The determination coefficients are 0.961 (60 s), 0.991 (300 s), 0.984 (1200 s), and 0.973 (1900 s), respectively. It confirms the applicability of the penetration model to the resin-embedded mouse brain. The CR speed and fluorescence intensity gradient at any time could be calculated by the penetration model. The calculated curves are shown in Fig. 3(g), and the vertical coordinate is the thickness of recovered fluorescence intensity at different percentages. According to the slopes of the curves, the CR speed is fast at the beginning and gradually slows down later. According to the fluorescence intensity gradient from the surface of the sample to the nether layers, the thickness of 50% fluorescence intensity ($Z_{0.5}$) is bigger than that of 100% fluorescence intensity ($Z_{1.0}$) at any time. The area below the $Z_{1.0}$ curve represents the region where the fluorescence recovers 100%, and the area above the $Z_{0.5}$ curve represents the region in the nether layers where the fluorescence recovers less than 50%.

Finally, as shown in Table 1, we obtain the diffusion coefficients and saturation coefficients of the Thy1-EGFP mouse brain embedded in several commonly used resins. Based on the coefficients, the curves in Fig. 4 are plotted to compare the CR thickness ($Z_{0.5}$) increasing with time. It can be found that the GMA resin-embedded mouse brain has the highest rate (red-dashed line), followed by London resin (LR) white (Structure Probe Inc.) resin-embedded mouse brain (blue-dashed line), and the Lowicryl HM20 resin-embedded mouse brain has the lowest rate (green-dashed line). The reasons are discussed as follows. First, the alkaline buffer is an aqueous solution; therefore, its diffusion coefficient in hydrophilic resin is higher than that in hydrophobic resin. Second, the diffusion coefficient of noncross-linking resin is usually higher than that of cross-linking resin, because the cross-linking resin has smaller micropores, lower porosity, and larger tortuosity factor.^12^^,^^16^ Therefore, a GMA, hydrophilic and noncross-linking resin has the largest diffusion coefficient and saturation coefficient; an LR white, hydrophilic and cross-linking resin has values smaller than those of GMA; and a Lowicryl HM20, hydrophobic and cross-linking resin has the smallest values.

**Table 1:** Diffusion and saturation coefficients of different samples.

Sample	D (${\mu m}^{2}/s$)	K
GMA resin-embedded mouse brain	0.332	2.227
LR white resin-embedded mouse brain	0.156	1.635
HM20 resin-embedded mouse brain	0.026	1.404

**Fig. 4:**
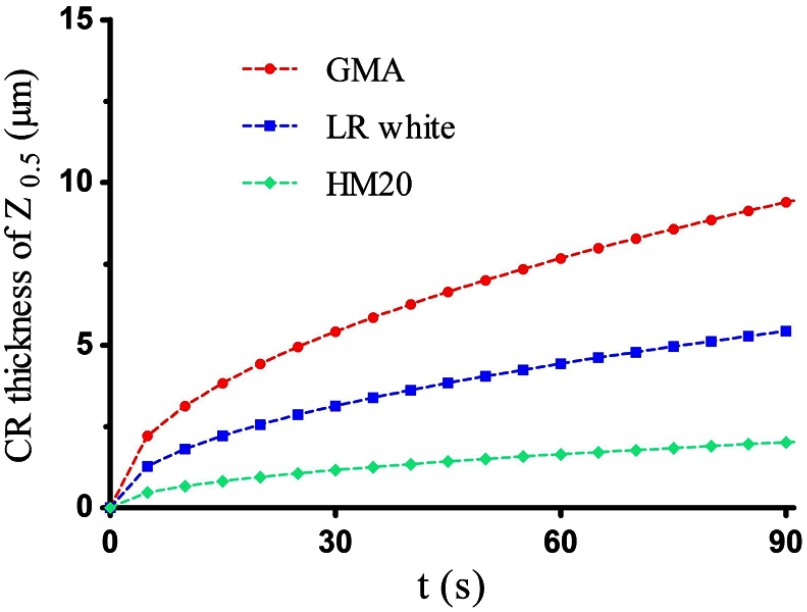
CR thickness ($Z_{0.5}$) increases with time in different samples. The penetration rate of the GMA resin-embedded mouse brain (red-dashed line) is the highest, followed by that of the LR white resin (blue-dashed line), and the Lowicryl HM20 resin has the lowest rate (green-dashed line).

In summary, we constructed a reliable penetration model to quantify the penetration rate in the resin-embedded specimen, based on Fick’s second law and the penetration data. This model, thus, gives a valuable theoretical explanation of the CR method and aids in optimizing the system parameters for mapping resin-embedded GFP-labeled biological samples.
